# Design of Mobile Health Tools to Promote Goal Achievement in Self-Management Tasks

**DOI:** 10.2196/mhealth.7335

**Published:** 2017-07-24

**Authors:** Brad Edward Dicianno, Geoffrey Henderson, Bambang Parmanto

**Affiliations:** ^1^ Department of Physical Medicine and Rehabilitation School of Medicine University of Pittsburgh Pittsburgh, PA United States; ^2^ Department of Rehabilitation Science and Technology School of Health and Rehabilitation Sciences University of Pittsburgh Pittsburgh, PA United States; ^3^ Department of Health Information Management School of Health and Rehabilitation Sciences University of Pittsburgh Pittsburgh, PA United States

**Keywords:** goals, self-care, mobile health, rehabilitation, smartphone, spinal cord injury, spinal dysraphism

## Abstract

**Background:**

Goal-setting within rehabilitation is a common practice ultimately geared toward helping patients make functional progress.

**Objective:**

The purposes of this study were to (1) qualitatively analyze data from a wellness program for patients with spina bifida (SB) and spinal cord injury (SCI) in order to generate software requirements for a goal-setting module to support their complex goal-setting routines, (2) design a prototype of a goal-setting module within an existing mobile health (mHealth) system, and (3) identify what educational content might be necessary to integrate into the system.

**Methods:**

A total of 750 goals were analyzed from patients with SB and SCI enrolled in a wellness program. These goals were qualitatively analyzed in order to operationalize a set of software requirements for an mHealth goal-setting module and identify important educational content.

**Results:**

Those of male sex (*P*=.02) and with SCI diagnosis (*P*<.001) were more likely to achieve goals than females or those with SB. Temporality (*P*<.001) and type (*P*<.001) of goal were associated with likelihood that the goal would be achieved. Nearly all (210/213; 98.6%) of the fact-finding goals were achieved. There was no significant difference in achievement based on goal theme. Checklists, data tracking, and fact-finding tools were identified as three functionalities that could support goal-setting and achievement in an mHealth system. Based on the qualitative analysis, a list of software requirements for a goal-setting module was generated, and a prototype was developed. Targets for educational content were also generated.

**Conclusions:**

Innovative mHealth tools can be developed to support commonly set goals by individuals with disabilities.

## Introduction

Goal-setting within rehabilitation is a common practice ultimately geared toward helping patients make functional progress. Goal-setting has been explored within many different conditions relevant to rehabilitation. For example, it has been used in pediatric rehabilitation [1], acquired conditions in adults such as traumatic brain injury [2], and older adults with conditions like dementia [3]. Although theories behind a variety of approaches to goal-setting date back to the 1970s, Schut and Stam are often credited for identifying the many positive benefits of goal-setting within rehabilitation [4,5] based on their published findings in 1994. A systematic review article also revealed that in order to be most effective, goal-setting activities should be patient-centered and involve collaboration between patients and clinicians [6]. It is a generally accepted concept that engaging patients in the goal-setting process is important and beneficial to psychosocial [7] and functional [8] outcomes, but unfortunately it is not always standard practice to have a patient-centered approach [4]. Reasons why clinicians may not engage patients are numerous and include lack of clinician education on how to engage patients, processes or tools that simply do not seek patient input, or lack of patient interest in participating [8]. However, in the cases when patients decline interest in participating, it is often because they may not understand their critical role in the process and are often more motivated to participate if they are informed of the value of their input and efforts are made to engage them [8].

Motivation of patients can be critical in promoting and maintaining behavioral change. According to self-determination theory (SDT), a theory of motivation that has been applied to multiple domains, including the health domain, several key psychological needs individually and cumulatively promote behavioral change [9,10]. These needs are autonomy (the belief that one’s behavior is self-originated and volitional), competence (the belief that one’s behavior is effective), and relatedness (the belief that one is cared for by others) [9,10]. Supporting these psychological needs has been shown to predict and produce increased motivation for behavioral change and help maintain behavioral change [9].

Over 20 years after Schut and Stam’s landmark article, controversy still exists over the most efficacious tools for increasing motivation and maintaining behavior change. The Functional Independence Measure is a well-known tool widely used in inpatient rehabilitation facilities and is used to track progress with functional activities [11]. However, its lack of customizability limits its ability to be used for the many activities people perform that fall outside of its standardized domains. One systematic review [12] suggested that Goal Attainment Scaling is an effective measure in adults, but it requires considerable time and training to administer. The Patient Specific Functional Scale (PSFS) has become popular due to its simplicity of use and applicability over a wide range of conditions, making it more practical to administer in a clinical setting [13].

We used our own approach to goal-setting within a wellness intervention consisting of a nurse case manager and an evidence-based protocol for the treatment and prevention of secondary conditions in individuals with spina bifida (SB) and spinal cord injury (SCI) [14]. In this study, 50 patients set up a goal tracking program and created short term (6 months to 1 year), long term (1 to 2 years), and maintenance (more than 2 years) health goals in collaboration with the nurse. Significant improvements were detected in health and patient experience of care outcomes, and almost 90% of goals were achieved. We also developed an mHealth tool, called the Interactive Mobile Health and Rehabilitation (iMHere) system, which consists of a mobile phone app for the patient and a Web-based portal used by the clinician [15]. The app has several modules for managing medical issues (medication management, bowel management, bladder management, skin integrity, and mood), which provide prompts to the patient to conduct self-management activities and allow them to send information, including photographs, to clinicians to alert them of medical issues. The clinician can use the Web-based portal to monitor a cohort of patients and triage medical issues that need to be addressed quickly. Input from over 120 patients, caregivers, and clinicians was formally obtained in sequential studies [16,17], through focus groups and formal in-laboratory testing that used validated methods of assessing usability. We also conducted a series of accessibility studies [18,19] to inform development for individuals with motor, cognitive and sensory impairments. We then conducted a clinical trial of the system in a group of adults with SB and found that higher utilization of the mHealth system resulted in improved self-management outcomes [20]. Additionally, a personal health record module and a module that educates users on issues relevant to their health have been subsequently built. We had not, however, incorporated a module into the app to help users with goal-setting specifically.

The use of electronic tools has been suggested as a way to effectively engage patients in goal-setting [8]. mHealth, for example, is a growing field that offers many approaches for patients to engage in self-management activities. Goal-setting and goal-attainment have actually been found to be motivators for using mHealth devices [21]. However, the bulk of mobile phone apps on the market are designed for helping the user manage one specific health or wellness activity such as weight loss or exercise, or one specific health condition such as diabetes. Few mobile phone apps engage the user into the types of goal-setting activities that are important for patients with disabilities who have very complex self-management routines. For example, patients with SB often need to adhere to a medication regimen, a bladder catheterization schedule, a bowel program, and a schedule to check skin for pressure ulcers, all while handling a host of other medical and psychosocial issues that may be impacting their lives [22].

Our ultimate goals in this study were to:

1. Qualitatively analyze the goals set by patients with SB and SCI in a wellness program to generate a list of software requirements for a goal-setting module within iMHere. Specifically, to accomplish this task we wished to answer several research questions:
  - What functionalities would be needed in a goal-setting module in order to support the various types of goals that people with SB or SCI desire to achieve?
  - What are the most common themes that describe the goals that people with SB and SCI set for themselves?
  - Is achievement of goals related to patient factors or characteristics of the goals?
2. Design a basic goal-setting module within iMHere, based on the PSFS.
3. Identify the educational needs of our target population to determine what content is important for the educational module within iMHere.

## Methods

This study was approved by the University of Pittsburgh Institutional Review Board. All research participants signed informed consent in order to participate. This study was a subcomponent of a larger, parent study reported previously in more detail [14]. Briefly, a wellness program was delivered to individuals with SB and SCI at an academic hospital-based outpatient physiatry clinic that partnered with an insurance division within an integrated health care delivery and financing system. Participants were recruited by the insurer and by their physicians. The program consisted of evidence-based guidelines, case management provided by a full-time nurse, and patient education. No mHealth tools were used in the parent study; all care was received in-person through the nurse at home visits or in an outpatient clinic. All participants were asked to create a wellness plan with help from the nurse. The wellness plan consisted of five short-term goals (6 months to 1 year), five long-term goals (1-2 years), and five maintenance goals (longer than 2 years). Participants were encouraged to set goals that were personally relevant, specific, measurable, and attainable [4,5]. Participants were also asked to meet quarterly for 2 years to evaluate progress toward their goals. Goal achievement was tracked by the nurse using a spreadsheet and checklists. Participants earned one gift card for establishing a wellness plan, one for achieving 80% of the short-term goals, one for achieving 80% of long-term goals, and one for achieving 80% of maintenance goals. Each gift card was worth US $25.

After the study was complete, all goals set by participants were first reviewed by one investigator and coded according to *goal temporality* (short-term, long-term, and maintenance), *goal theme* (the general wellness topic the goal addressed), *goal type* (the method by which data were recorded to help a participant track progress toward a goal), and *goal achievement* (whether or not the goals were reached by the end of the study). The same investigator reviewed and recoded the data a second time while blinded to the results of the first review. Final codes were assigned to goals if the two ratings were the same. This occurred in 710 of the 750 (94.7%) goals. In 40 goals, there was a discrepancy noted in goal theme. To resolve the discrepancy, a second investigator then independently coded the 40 items on which there was disagreement within the ratings of the first investigator. The second investigator was blinded to the review of the first investigator. These remaining 40 goals were then assigned a final code based on agreement between codes of the two separate investigators. In addition, the types of gift cards that participants selected were recorded.

A Cronbach alpha level of *P*<.05 was selected a priori. SPSS version 24 (IBM Corp) was used to test for associations among categorical variables. To evaluate whether there was any significant difference in achievement of goals based on sex, goal temporality, goal theme, and goal type, chi-square analyses were used. For the analysis involving goal theme, only the seven most common themes (as designated by number of goals) and “other” were analyzed.

## Results

A total of 69 individuals with SB and SCI were consented; 4 were excluded for failing to meet inclusion criteria or withdrawing before baseline data could be collected. The remaining 65 participants enrolled in the intervention. Of those 65 participants, 50 completed a wellness plan. Goal data from these 50 participants who completed a wellness plan were included in this study. A total of 24 participants (48%) were female; 26 participants (52%) were male. Furthermore, 37 (74%) had SB; 13 (26%) had SCI. Average age was 38.7 (SD 14.1) years. Additional demographic data obtained from the parent study can be found in the prior publication [14].

### Goal Temporality

Fifteen goals (5 short term, 5 long term, and 5 maintenance goals) from each of the 50 individuals were analyzed, for a total sample of 750 wellness goals.

### Goal Theme

Of the 750 wellness goals, 15 themes were identified. Table 1 displays the themes in more detail.

### Goal Type

Based on the types of goals that participants selected, investigators identified three methods that were used to help participants and the nurse track progress toward achieving those goals.

1. Checklist: 24% of goals involved a simple checklist indicating whether or not the event or aim was achieved. This type of goal would need to be manually “checked off” the list.
  - A single task that occurs at one point in time (eg, scheduling an appointment with a neurologist).
  - A task that begins but then is an ongoing event after the task is accomplished. The goal focuses on the initiation of the task, not monitoring continued compliance with the task (eg, beginning to wear wrist splints for carpal tunnel syndrome).
  - A goal that is focused on an outcome with no specific date attached (eg, achieve employment, or stop smoking).
  - A goal focused on the initiation of a behavior (eg, begin to seek employment).
  - Achieving a situation in which the person is “free of a certain condition” (eg, achieve intact skin, free of pressure ulcers). This type of goal was discouraged because it may be difficult to achieve even if a participant is compliant with treatment, but nonetheless, participants chose some of these goals.
  - A situation that arises only under certain circumstances (eg, if skin breakdown occurs, participant will notify a clinician).
  - “As needed” goal (eg, participant will take a medication for pain when it is needed).
2. Data-tracking: 48% of goals involved recording data sequentially over repeated time intervals. This type of goal would be achieved if the value of the data fell within a certain range, achieved a threshold value, or reached a threshold by a specific date.
  - Achieving or maintaining certain health parameters (eg, goal to achieve a target weight or maintain blood pressure within specific parameters).
  - Achieving or maintaining a certain goal for health activities (eg, intake a specific amount of fruits, vegetables, fluid, protein, or sodium).
  - Achieving a degree of compliance with self-management activities that occur at a certain frequency (eg, skin checks, exercise regimen, medications, appointments, or bladder or bowel program).
  - Achieving a specific goal by a certain date (eg, reach target weight by the participant’s birthday).
3. Fact-finding: 28% of goals involved recalling specific facts or finding information about topics that can impact health, promote health outcomes, and prevent secondary complications.
  - Recalling specific knowledge (eg, being able to identify the signs and symptoms of skin breakdown or urinary tract infections, foods that are more appropriate in diabetes, or foods low in sodium).
  - Seeking a reference that will serve as a blueprint for future action (eg, finding an exercise program that can be performed while in a wheelchair, or finding information about housing).

Table 2 displays more detail about how goal theme was related to gender, goal type, and goal classification. Table 3 displays the themes of the fact-finding goals which identify the educational needs of our target population and determine what content is important for the educational module within iMHere.

**Table 1:** Goal themes.

Goal theme	Description of theme	Total number	Proportion of total goals (%) (n=750)
Diet	Improve or maintain caloric intake, make good food choices, or reach a weight loss goal through diet	108	14.4
Bladder/Bowel	Maintain or improve continence, recognize important signs or symptoms, adhere to a prescribed regimen for bowel or bladder care or appointments	102	13.6
Exercise	Improve or maintain physical activity amount, quality or frequency, or to reach a weight loss goal through exercise, adhere to prescribed physical therapy regimen, identify adaptive exercises	100	13.3
Skin	Be more compliant with prescribed skin care regimens or proper use of wheelchair equipment with the goal of preserving skin integrity or healing an open wound	95	12.6
Appointments	Track and maintain or improve adherence to medical appointments	64	8.5
Other/Medical	Adhere to care prescribed by primary care physician, gynecologist or other specialist, manage lymphedema	55	7.3
Medications	Track and maintain or improve adherence to medication schedule, learn about medications	52	6.9
Equipment	Maintain, acquire, or properly use assistive technology and orthoses for mobility or self-management	48	6.4
Other/Non-Medical	Participate in home and community activities	39	5.2
Work/School	Acquire or maintain employment, participate in vocational rehabilitation or school activities	23	3.1
Cardiovascular risk factors	Blood pressure and diabetes control, smoking reduction/cessation	16	2.1
Mood/Sleep	Identify methods or coping mechanisms to improve mood or restful sleep, follow recommendations of psychologist or psychiatrist, practice better sleep hygiene	14	1.9
Weight loss	Achieve a desired target weight without specifying the plan to do so	14	1.9
Pain	Prevent, reduce or maintain pain thresholds or be compliant with prescribed pain regimens	12	1.6
Driving/ Transportation	Acquire or properly use assistive technology for driving or transportation, acquiring a license or skills needed to drive	8	1.1

**Table 2:** Detailed display regarding how goal theme was related to gender, goal type and goal classification.

Goal theme		Sex		Goal temporality				Goal type			
	Total number	Female	Male		Short-term	Long-term	Maintenance		Data tracking	Fact finding	Checklist
Diet	108	51	57		46	48	14		49	57	2
Bladder/Bowel	102	50	52		45	19	38		29	43	30
Exercise	100	50	50		23	42	35		70	26	4
Skin	95	38	57		59	12	24		24	38	33
Appointments	64	34	30		8	5	51		59	0	5
Medications	52	28	24		6	2	44		47	3	2
Equipment	48	20	28		15	28	5		25	6	17
Other	181	89	92		48	94	39		54	40	87
Total	750 (100%)	360 (48%)	390 (52%)		250 (33%)	250 (33%)	250 (33%)		357 (48%)	213 (28%)	180 (24%)

**Table 3:** Themes of the fact-finding goals which identify educational needs.

Theme	Details
Skin integrity	Signs and symptoms of skin breakdown and wound infection; areas at risk for skin breakdown; how to prevent skin breakdown; importance of position changes; equipment affecting skin breakdown
Bowel/bladder	Signs and symptoms of urinary tract infections; basics of a bowel program; benefits of a bowel program
Shunts	Signs and symptoms of shunt malfunction
Medication	Identify the indication for each medication
Diet	Basics of good nutrition; nutrition foods; information on “my healthy plate” or food pyramid; proper portion sizes or portion control; foods to help meet the daily energy requirement; recommendations on fluid intake; whole and wholesome foods versus junk foods or empty calories; nutritious foods high in protein to promote wound healing
Exercise	Benefits of exercise; how to start an exercise program; exercises that can be performed in a wheelchair; exercises that can be performed at home
Sleep	Sleep hygiene; techniques to promote restful sleep
Pain	Alternatives to pain medications; techniques to promote pain control; common over-the-counter medications for pain
Weight control	Benefits of weight control; risks of being overweight
Mood	Relaxation techniques; techniques to improve mood; coping techniques
Smoking	Effects of smoking on health and on circulation; effects of smoking on skin breakdown or wound healing

### Goal Achievement

Of the 750 total wellness goals, 669 (89%) were achieved; 81 (11%) were not achieved. Furthermore, 20 out of 50 participants (40%) achieved all of their goals, and 42 participants (84%) achieved at least 13 out of 15 goals (87%). Eight individuals accounted for 67% of the goals that were not achieved. There was a significant difference in achievement of goals based on sex (*P*=.02), as well as diagnosis, goal temporality, and goal type (*P*<.001 for each respective analysis). Those of male sex and with SCI diagnosis were more likely to achieve goals than females or those with SB. Table 2 indicates the number of times a theme was reported across sex, goal temporality, and goal type.

Shorter temporality of the goal coincided with a higher likelihood that the goal would be achieved. Almost all goals surrounding fact-finding were achieved. There was no significant difference in achievement based on goal theme (*P*=.75; See Table 4).

**Table 4:** Achievement of goals according to various factors.

Factor		Achieved		Total number		Percent achieved (%)		Not achieved	
**All goals**		669		750		89		81	
**Sex** (*P*=.02)								
	Female (24 people)		311		360		86		49
	Male (26 people)		358		390		92		32
**Diagnosis** (*P*<.001)								
	SB (37 people)		479		555		86		76
	SCI (13 people)		190		195		97		5
**Goal Temporality** (*P*<.001)								
	Short-term		246		250		98		4
	Long-term		222		250		89		28
	Maintenance		201		250		80		49
**Goal Theme** (*P*=.75)								
	Diet		102		108		94		6
	Bladder/Bowel		93		102		91		9
	Exercise		89		100		89		11
	Skin		87		95		92		8
	Appointments		58		64		91		6
	Medications		45		52		87		7
	Equipment		43		48		90		5
	Other		152		181		84		29
**Goal Type** (*P*<.001)								
	Data tracking		312		357		87		45
	Fact finding		210		213		99		3
	Checklist		147		180		82		33

### Incentives

Participants chose gift cards as incentives for achieving goals. The most popular preference was for healthy dining or groceries at 41.2% of participants, followed by 27.7% opting for entertainment, 12.4% for general retail, and 11.3% for clothing or personal care. Only 4.5% of participants chose gift cards catering to home improvement, and 2.8% chose gift cards for gasoline.

### Operationalization of Findings Into Software Requirements

Based on the cumulative results, a list of software requirements was developed:

- Patient can select a goal functionality (checklist, data tracking, or fact-finding) when creating a goal
- Patient can generate a checklist of multiple goals
- Patient can choose from a default list of common goals or create his or her own goals
- Patient can record whether a goal was achieved or is in progress
- Patient can self-report progress toward achieving each goal
- App can auto-populate goals with data to show progress in goal-achievement
- Patient can record goals that are related to obtaining and understanding educational material
- Caregiver, peer or clinician can view goals of a patient and provide encouragement
- Caregiver, peer or clinician can suggest goals to patient
- Patient can choose designees who are able to access and modify goals
- Goals should be linked to deadlines or calendars
- App should provide tips on goal-setting

### Basic Prototype of Goal-Setting Module

**Figure 1:**
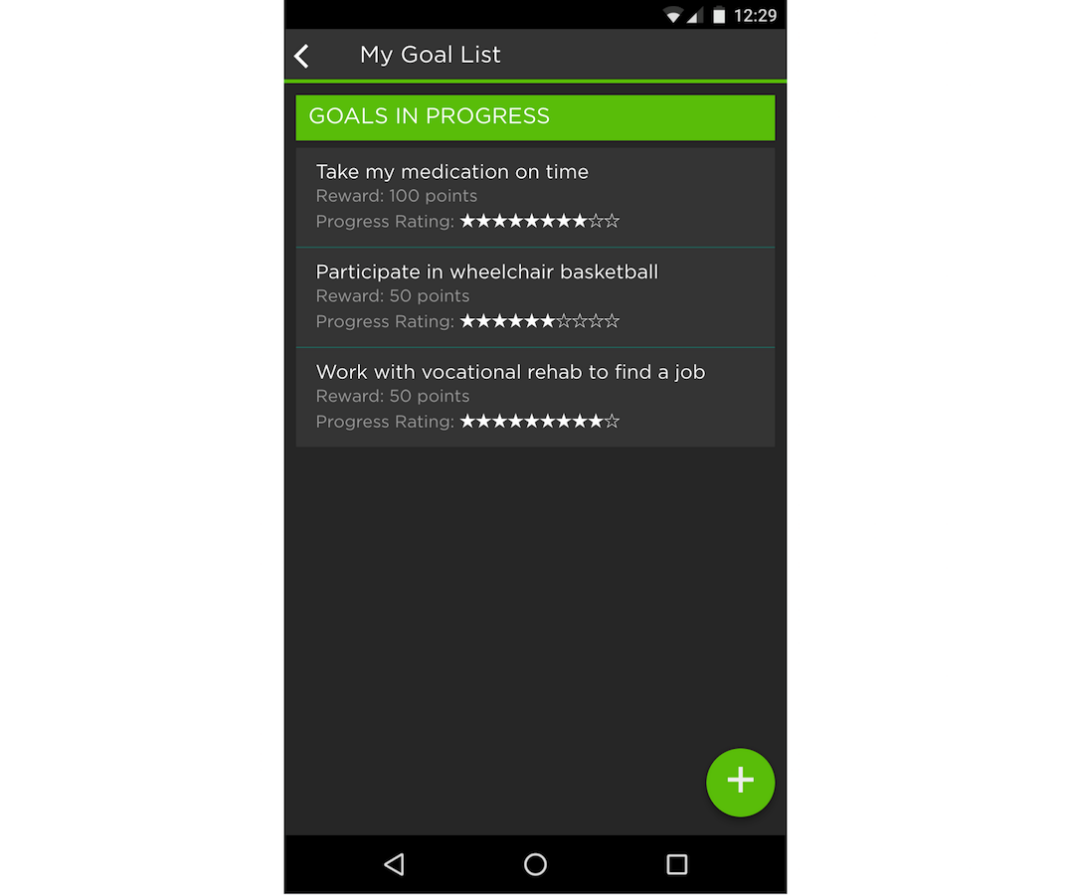
Prototype of goal-setting module for iMHere.

A goal-setting module was created for iMHere, based on the PSFS (see Figure 1).

## Discussion

### Principal Findings

To our knowledge, this is the first study to examine patient goals in an effort to develop more robust mHealth tools that support goal achievement in self-management. We successfully developed a goal-setting module based on the PSFS. We also identified three functionalities that may possibly help users track and achieve goals when using mHealth systems.

About one-quarter of the goals required the functionality of a checklist. The checklist is a simple tool that has been shown to produce improved outcomes in a number of health-related and other disciplines [23]. This type of goal-setting feature is already available in the current version of iMHere. However, more complex functionality, such as the ability to add sub-items to lists, will be added to support complex tasks that require multiple steps.

Approximately half of the goals required ongoing tracking of progress toward meeting that goal. On the PSFS, patients are asked to rate on a scale how well they subjectively feel they are accomplishing their goal. However, within mHealth systems, it is now possible to collect, analyze, and display summary data back to the user as a way to “automate” the tracking of progress toward a goal. Data collected and displayed in this manner can be self-reported data or objectively gathered data. For example, in the current version of iMHere, users are able to report on a regular basis whether they have been taking each of their medications. In future versions of iMHere, data on self-reported compliance with medications will be displayed back to the users as a bar graph to indicate how often the users indicated they took medications over a period of a week or a month. Objectively gathered data can also be collected from peripheral devices. For example, activity monitors connected to future versions of iMHere will be used to show users how well they are meeting a physical activity goal.

The remaining goals involved recalling specific facts or finding information about health topics. Tracking achievement of a fact-finding mission can be accomplished with a checklist. Because almost all fact-finding goals were achieved, it is possible that participants chose fact-finding goals that were “easy” to achieve; therefore, more complex checklists or other functionalities may be needed if a patient would choose a more complex fact-finding goal that has multiple steps within it. Recall of specific information from materials accessed could be evaluated through a quiz. In the current version of iMHere, an educational module presents medical information relevant to a user’s health and also provides quizzes about that information. A future version of iMHere will link the educational quizzes to the goal-setting feature. A goal of our study was to identify the educational needs of our target population to determine what content is important for the educational module within iMHere. Based on the fact-finding goals set by participants, we will be building educational content to support themes identified in Table 3.

The data collected on themes of goals set by participants were useful in several ways. First, the data validated the importance of modules that have already been developed for iMHere (medication management, bowel management, bladder management, skin integrity, and mood). Second, the data generated development ideas for modules for other important health domains such as diet, exercise, weight management, appointment scheduling, tracking needs for items such as wheelchairs or adaptive driving, vocational and school activities, pain, and sleep. In this study, the theme with the largest representation of goals was diet. Therefore, it is not surprising that the gift cards participants selected for meeting those goals included options for healthy eating or purchasing groceries. Thus, a third concept that emerged was that specific incentives could be linked to types of goals achieved. Finally, data helped inform what types of goals could be included as “default” goals from which patients can select.

Achievement of goals was related to several factors. Individuals with SB and females achieved fewer goals than those with SCI and those of male sex. It may be that those with SB or females set goals that were lofty and therefore more difficult to achieve, they were less motivated to achieve their goals, or they had greater medical complexity, making goal achievement somewhat more difficult. More work is needed on larger samples to understand the factors that contributed to goal achievement. It was not surprising that short-term goals were achieved more often than long-term goals, since the participants tended to set more easily achievable activities as the short-term goals. Despite having some degree of impairments in executive function [16], individuals with SB did not seem to have difficulty conceptualizing or setting goals, an encouraging finding considering the majority of individuals with SB in this study had hydrocephalus.

Based on the variety of goals set and lessons learned from the clinical trial, we also identified other features that are important in creating goal-setting features in mHealth. First, a clear need exists for patients, clinicians, and even caregivers to be involved in the goal-setting process. Caregiver goals may be quite different from the goals of the persons they assist [24]. As a result, we plan to create a goal-setting module that can be viewed and edited through role-based access via both patient and caregiver apps, as well as a clinician portal. Additionally, we plan to provide “free text” options to allow for custom goals to be written, as well as “quick select” options for goals that are commonly set and which can be tracked with automated features within the app. Finally, it may also be useful to display “socially persuasive” data to users as a way to motivate them to achieve goals. For example, it may be possible to display a user’s compliance with taking medications in reference to the average compliance of peers. This technique has been found in other studies to increase the likelihood of achieving desired health outcomes [25].

The results of this study inform the software requirements for the graphical user interface of the iMHere goal-setting module. Several design changes will be made in order to meet these requirements. First, when entering a new goal, the patient will be able to: (1) select a goal functionality (checklist, data tracking, or fact-finding), which will determine how the app will track user progress toward achieving the goal, (2) choose one or more individuals (eg, user, clinician, caregiver) who will be responsible for independently determining whether the goal was achieved, and (3) choose supporters (eg, caregivers or peers) who are able to provide encouragement. Second, the goals will be marked as “in progress” by default until they are marked as “achieved” by a patient or his or her designee. Third, patients or designees will be able to enter a goal by typing as free text or choosing from a list of common “default” goals categorized by themes in Table 1. Fourth, each goal will contain an option to enter a deadline for the goal (selected from a calendar, or alternately, selected by designated time frame, such as one month) and options to receive prompts that query the patient whether he or she is on track to achieving the goal (eg, prompts appear on recurring schedule, or after a period of time, such as 75% of time has passed). Fifth, when first using the module or when accessing instructions, patients and designees will be reminded to create goals that are personally relevant to the patient, specific, measurable, and attainable [4,5].

### Limitations

A few limitations of this study deserve discussion. Our sample was limited to SB and SCI. External validity of this study is therefore limited to these populations. However, some findings may be relevant or translatable only to those particularly interested in engaging in health promotion activities. Our sample was also small; however, due to the large number of goals set by each participant, a large amount of goal-setting data was collected. One limitation of the parent study is that it lacked a control group. Because it was a clinical program, it was not possible or ethical to randomize participants into a group that did not receive these services. We therefore designed the parent study as a cohort trial and used intention to treat analysis, given the increasing popularity of integrated delivery systems and clinical utility of pragmatic research designs.

### Future Directions

In future work, more complex functionalities could be added to the software, such as stakes (eg, consequences for not achieving the goal such as peers being notified), suggestions for brainstorming barriers (eg, option to contact clinician for help, extend deadline, or delete goal), and ability to opt into participating in socially persuasive group challenges, or pre-built goal modules that are designed around best practices (eg, workflow that guide the user how to conduct an evidence-based pressure ulcer prevention program). We plan to study the goal-setting module with patient, caregiver, and clinician users in future usability and feasibility trials.

In conclusion, lessons learned from analyzing wellness goals of participants with SB and SCI have been distilled into recommendations intended to help spearhead future development of iMHere. Goal-setting features in mHealth apps such as iMHere may be able to aid individuals in creating, pursuing, and achieving their wellness goals.

## Abbreviations

iMHere: Interactive Mobile Health and Rehabilitation
mHealth: mobile health
PSFS: patient specific functional scale
SB: spina bifda
SCI: spinal cord injury
SDT: self-determination theory
